# Magnetic resonance imaging-guided intracranial resection of glioblastoma tumors in patient-derived orthotopic xenografts leads to clinically relevant tumor recurrence

**DOI:** 10.1186/s12885-023-11774-6

**Published:** 2024-01-02

**Authors:** Anais Oudin, Pilar M. Moreno-Sanchez, Virginie Baus, Simone P. Niclou, Anna Golebiewska

**Affiliations:** 1 NORLUX Neuro-Oncology Laboratory, Department of Cancer Research, Luxembourg Institute of Health (LIH), 6A, Rue Nicolas-Ernest Barblé, Luxembourg, L-1210 Luxembourg; 2 Department of Life Sciences and Medicine, Faculty of Science, Technology and Medicine (FSTM), University of Luxembourg, Belvaux, L-4367 Luxembourg

**Keywords:** Glioblastoma, Patient-derived orthotopic xenograft, Brain surgery, Tumor resection, In vivo imaging

## Abstract

**Background:**

Preclinical in vivo cancer models are essential tools for investigating tumor progression and response to treatment prior to clinical trials. Although treatment modalities are regularly assessed in mice upon tumor growth in vivo, surgical resection remains challenging, particularly in the orthotopic site. Here, we report a successful surgical resection of glioblastoma (GBM) in patient-derived orthotopic xenografts (PDOXs).

**Methods:**

We derived a cohort of 46 GBM PDOX models that faithfully recapitulate human disease in mice. We assessed the detection and quantification of intracranial tumors using magnetic resonance imaging (MRI).To evaluate feasibility of surgical resection in PDOXs, we selected two models representing histopathological features of GBM tumors, including diffuse growth into the mouse brain. Surgical resection in the mouse brains was performed based on MRI-guided coordinates. Survival study followed by MRI and immunohistochemistry-based evaluation of recurrent tumors allowed for assessment of clinically relevant parameters.

**Results:**

We demonstrate the utility of MRI for the noninvasive assessment of in vivo tumor growth, preoperative programming of resection coordinates and follow-up of tumor recurrence. We report tumor detection by MRI in 90% of GBM PDOX models (36/40), of which 55% (22/40) can be reliably quantified during tumor growth. We show that a surgical resection protocol in mice carrying diffuse primary GBM tumors in the brain leads to clinically relevant outcomes. Similar to neurosurgery in patients, we achieved a near total to complete extent of tumor resection, and mice with resected tumors presented significantly increased survival. The remaining unresected GBM cells that invaded the normal mouse brain prior to surgery regrew tumors with similar histopathological features and tumor microenvironments to the primary tumors.

**Conclusions:**

Our data positions GBM PDOXs developed in mouse brains as a valuable preclinical model for conducting therapeutic studies that involve surgical tumor resection. The high detectability of tumors by MRI across a substantial number of PDOX models in mice will allow for scalability of our approach toward specific tumor types for efficacy studies in precision medicine-oriented approaches. Additionally, these models hold promise for the development of enhanced image-guided surgery protocols.

**Supplementary Information:**

The online version contains supplementary material available at 10.1186/s12885-023-11774-6.

## Background

Preclinical cancer modeling, including recapitulation of various steps of therapeutic interventions applied to patients, has been an extensive area of research. Spontaneous tumors in animals are rare [1–3]; thus, modeling solid tumors requires specific protocols, mostly applied in rodents. The development of tumors in vivo requires chemical or genetic engineering of rodent cells, allowing for the growth of tumor cells of the same origin as the host. As testing therapeutic interventions often require models displaying molecular features resembling fully developed patent tumors, xenografts based on implantation of patient-derived tumor cells (i.e., patient-derived xenografts (PDXs) or patient-derived orthotopic xenografts (PDOXs)) are more valuable [4, 5]. Mice are more commonly used than rats due to easier maintenance, higher experimental throughput and a large range of transgenic strains representing immunocompromised backgrounds. Although the majority of patients with aggressive tumors undergo surgery as part of the standard-of-care treatment, this step is rarely recapitulated in the preclinical setting. Surgical resection has been applied to easily accessible or nonvital organs such as breast or bone to assess the metastatic potential of xenografted tumor cells [6, 7]. Assessing tumor recurrence after surgery at the original site is more challenging due to the small size of the organs in mice, low resectability area without damaging the organ, limited integration of the xenografted tumor into the organ or the difficulties of primary tumor assessments [8, 9]. Elaborated and repeatable animal experimentation protocols are needed to envisage the application of resection in preclinical settings at a large scale.

Surgical resection is the first-line treatment for aggressive brain tumors. Diffuse gliomas are the most common primary brain tumors, including oligodendrogliomas and astrocytomas of different grades as well as the most aggressive grade 4 glioblastomas (GBMs) [10]. Diffused gliomas are characterized by dispersed growth of tumor cells in the brain with no clear tumor demarcation, leading to incomplete resection during surgery and inevitable tumor recurrence. Nevertheless, maximal safe surgical tumor resection of the MRI-detectable contrast-enhancing zone is the crucial step of standard-of-care treatment: it removes the bulk of the tumor, reduces brain pressure, improves neurological function recovery and allows for accurate diagnosis by histopathology. While certain brain locations and limited detection of infiltrative tumors by magnetic resonance imaging (MRI) prevent complete resection of GBMs, it has been shown that the extent of resection, including contrast-enhanced and noncontrast-enhanced regions, is correlated with better survival [11]. Resection in the brain can be improved with image-guided surgery protocols, including intraoperative MRI [12], awake craniotomy for cortical mapping [13], confocal intraoperative microscopy, fluorescence-guided surgery with 5-aminolevulinic acid (5-ALA) [14] and intraoperative mass spectrometry [15]. As radiotherapy and chemotherapy usually follow surgical resection, recapitulating tumor removal may be crucial for testing the efficacy of treatments in a clinically relevant setting. It remains to be seen to what extent these protocols can be recapitulated in preclinical models.

Brain tumor surgery protocols in preclinical models are scarce and thus far have mostly been applied in xenografts derived from long-term in vitro cell cultures, such as human U87 cells and mouse glioma GL261 and CT-2 A cells [16–19]. However, these models do not recapitulate well the histopathological features of patient tumors. The invasive capacities of majority of cell lines are very limited, which may impact the evaluation of resection coordinates, technical procedures during surgery and follow up readouts. Until now, protocols for the brain tumor resection in preclinical models have primarily relied on optical imaging techniques, specifically fluorescence and bioluminescence, for detecting tumor cells in rodent brains. Such protocols necessitate the genetic modification of tumor cells with a fluorescent or bioluminescent marker prior the implantation, thereby limiting their applicability to primary patient-derived models. Fluorescent agents such as 5-ALA can only support detection of a subset of tumor cells in the areas of leaky blood brain barrier and are applicable only to most aggressive gliomas [20]. While one protocol applied surgery on a GBM PDOX based on primary organoid cultures in nude rat brain [21], it remains to be seen if it is feasible in a larger cohort of PDOXs derived in mice.

Here, we show that MRI can be applied for tumor detection and quantification in a large number of primary glioma PDOX models developed in mice. An intracranial tumor resection procedure is possible in immunodeficient mice with developed primary diffuse tumors in the brain without extensive manipulation of the original tumor material. Similar to the preoperative neuronavigation applied in patient tumors, we were able to perform resection of the tumors with use of stereotactic frame following MRI-guided coordinates in PDOX models, which led to prolonged survival of mice. Surgical procedures guided by MRI images allow preservation of the invasive tumor zone, and the remaining GBM cells reform the tumor in the same tumor cavity with similar histopathological features. Our protocol closely mirrors the clinical scenario, facilitating its application in future preclinical studies that necessitate a combination of surgery and local or systemic therapeutics.

## Methods

### Glioma PDOX models

PDOXs were obtained by implantation of human glioma organoids in the mouse brain following our standard protocol [22]. Shortly, primary organoids were derived from mechanically digested tumor tissue. Tissue fragments were cultured for up to 2 weeks in non-adherent conditions at 37 °C under 5% CO2 and atmospheric oxygen in DMEM medium, 10% FBS, 2 mM L-Glutamine, 0.4 mM NEAA, and 100 U/ml Pen–Strep (all from Lonza). Organoids with a diameter of 300–1000 μm were selected for intracranial implantation. For regular maintenance of the PDOX models, we implanted tumor organoids (6 per brain) into the right frontal cortex of NSG (NOD. Cg-Prkdc^scid^ Il2rg^tm1WjI^/SzJ, Charles River Laboratories, France) mice, which allows for high tumor take of primary patient tumor tissue. The detailed protocol steps and molecular characterization are presented in [22, 23].

For this study, GBM organoids were implanted into nude mice (athymic female nude mice, Charles River Laboratories, France) at least one week after acclimatization in the animal facility. This allowed assessment of the resection protocol in the least immunocompromised mouse background in the animals of the same age and sex. Animals were housed in a specific pathogen-free (SPF) facility under a controlled environment (temperature, humidity, and light) with free access to water and food. Animals were sacrificed at the endpoint defined in the scoresheet.

### Tumor resection protocol

#### GBM organoid implantation

To assess the feasibility of the surgical resection for the first time in PDOXs, we selected a small sample size. The evaluation of number of mice was based on our previous experience in comparative studies, where a sample size power statistical analysis, using a two group chi squared test with a 0.05 two-sided significance level, revealed a prerequisite for 7 mice per group to give 82.2% power to detect a difference, assuming a Group 1 proportion, π^1^, of 0,85 and a Group 2 proportion, π^2^ of 0,15 (odds ratio of 0,031). Nude mice (10–11 per model) were implanted with P3 and T16 GBM organoids (6 per brain) under general anesthesia following our standard procedure [22]. Briefly, mice were anesthetized with ketamine (Nimatek, Dechra) and xylazine (Rompun, Bayer) during surgery followed by inhalation of 2% isoflurane (CP Pharma) and 100% oxygen thorough MRI via a nose cone adapter. The procedure was applied upon loss of hind-foot withdrawal and corneal reflex. A burr hole was drilled in the frontal cortex of the mouse brain. GBM patient-derived organoids were slowly injected using a Hamilton syringe (Hamilton), and tumor growth was followed weekly by MRI. Eight mice per model were planned for surgical resection, of which one mouse per model was sacrificed shortly after the surgery and seven were used for monitoring tumor recurrence. One mouse per model was sacrificed without surgery at the time point of resection to verify tumor growth. Two mice for P3 and one mouse for T16 were used as controls without surgical intervention until the endpoint. To assess the control survival arm of each PDOX model, historical survival data were extracted from additional PDOXs implanted at different time points (T16: n = 10 corresponding to the same organoid generation, P3: n = 8 corresponding to the same organoid generation and the same organoid batch). This allowed for reduced use of animals following 3R rules. Previous experiments were performed with the same experimental settings and mouse strain.

#### Surgical resection

To perform surgical resection, mice (n = 8 per model) were anesthetized again using ketamine 100 mg/kg (Nimatek) and xylazin 10 mg/kg (Rompun). To ensure sufficient analgesia during surgery, we performed a subcutaneous injection of Marcaine 0.25% Adrenaline (bupivacaine hydrochloride, adrenaline) 5 mg/kg into the scalp and a subcutaneous injection of Vetergesic (buprenorphine 0.1 mg/kg, Ecuphar) on the back. Mice were placed on the stereotactic frame (Narishige), and the skin was incised to expose the burr hole in the scalp from the tumor inoculation via the organoid implantation procedure. A square of approximately 3 mm of side length was burred with a micro drill of 0.7 mm diameter (FST) around the first injection site, the piece of bone was removed, and the dura mater was gently peeled using Bonn Micro probes (FST 10030-13). Bonn Micro probes were placed to define the border and the deepness of the resection site. The needle was gently moved in the brain to destroy the tumor-containing tissue before aspiration with a small glass Pasteur pipette. A piece of polypropylene mesh (Ethicon) was fixed with Histoacryl glue (Braun) to compensate for the lack of skull, and the skin was closed with Ethilon 3 − 0 suture (Ethicon) and cleaned with 7.5% Braunol (Braun). The mice were placed in a warm chamber (Pecoservice) until recovery from anesthesia. A subcutaneous injection of buprenorphine (0.1 mg/kg, Ecuphar) was repeated 6 h after surgery.

#### Animal welfare monitoring

The animal experimentation protocol was classified as “moderate”, as the pain from brain surgery was compensated by local and general analgesia. Mice were carefully monitored for 48 h for neurological adverse effects that could arise after brain surgery. Mice were observed and scored daily using a score sheet according to the different possible symptoms [24]. The score sheet included abnormal behavior (no nest, fighting), neurological symptoms (seizure, always turning), kyphosis, body weight, and wound aspect (infection, ichor, and early removal of stitches). Scores of 0 (no symptoms), 1 (symptoms start & body weight loss ≤ 5%), 2 (established symptoms & body weight loss ˃5% and ˂15%) and 3 (severe symptoms & body weight ≥ 15%) were applied according to daily observation, and mice were euthanized by cervical dislocation when 3 symptoms reached a score of 2 or one symptom reached a score of 3. Randomization and blinding were not feasible due to experimental procedures and visual/MRI-based detection of the surgical procedure. GraphPad Prism was used for survival analysis. Kaplan‒Meier survival curve differences were assessed by a log-rank test. A P value < 0.05 was considered significant.

### Magnetic resonance imaging (MRI)

#### In vivo

Mouse anesthesia was induced and maintained by 2.5% isoflurane in oxygen. Mice were placed in the 3T MRI scanner (MRsolutions), with breathing and temperature constantly monitored during the acquisition following our standard protocol for 6 min with the 23-mm volume coil setup [22]. A Fast Spin Echo T2-weighted MRI sequence defined with a field of view of 25 mm, matrix size of 256 × 256, TE of 68 ms, TR of 3000 ms, an average of 4, an echo train of 8 and slide thickness of 1 mm was used for multislice axial acquisition. Tumor growth was followed up by MRI weekly. Animals were anesthetized with an isoflurane/oxygen mixture (1–3%, CP Pharma) throughout the scan, with respiration and body temperature monitored via an MRI monitoring system.

#### Ex vivo

After mouse euthanasia, brains were excised and directly fixed in 4% paraformaldehyde (PFA, Sigma) for 5 days (P3) or 24 h (T16). A 15 ml tube containing brain tissue in 4% PFA was inserted into the head coil, and a Fast Spin Echo T2-weighted MRI sequence was applied for approximately 1 h with the following parameters: a field of view of 20 mm, matrix size of 240 × 256, TE of 51 ms, TR of 6000 ms, an average of 32, an echo train of 8 and slide thickness of 1 mm to increase the picture quality.

#### Image analysis

MRI data were analyzed with ImageJ for tumor quantification and Vivo Quant software for the coordinates of resection. Tumor volume was quantified as described previously [22] based on hyperintense tumor regions of the brain in T2-weighted axial MRI slices. Tumor volume in mm^3^ was defined by the sum of the tumor area on each slice (slice thickness is 1 mm). To establish coordinates for resection, tumor borders (top-bottom, left-right) were identified manually from the injection area using Vivoquant software.

### Immunohistochemistry

After euthanasia, mouse brains were extracted, fixed in 4% PFA (Sigma) and embedded in paraffin. Section (3 and 8 μm) were used for immunohistochemistry as previously described [23]. Human GBM cells were recognized by hematoxylin/eosin (Dako/Sigma respectively), as well as immunostaining with human-specific Nestin (Abcam AB6320; 1:500, 3 h incubation, room temperature). Additionally, actively proliferating tumor cells were stained with human-specific Ki67 (Dako M7240; 1:75, 1 h incubation, room temperature), and mouse-derived microglia/macrophages were detected with Iba1 (Biocare Medical CP290A; 1:1000, overnight incubation at 4 °C) followed by a 30 min incubation with secondary antibodies. Co-staining with hematoxilin was applied to detect tumor areas with high nuclei density when necessary. The signal was developed with the Envision + System/HRP Kit in 5–20 min (K4007, Agilent/Dako). Iba1^+^ cells were quantified based on the ImageJ plugin [25]. To enable sensitive detection and quantification of the size and density of blood vessels, CD31 (Cell Signaling Technology #77,699; 1:100, overnight incubation at 4 °C) staining was performed using an Opal 3-Plex Manual Detection Kit (Akoya Biosciences) following the manufacturer’s guidelines. Cell nuclei were counterstained with Hoechst 33,258 (1 µl/ml; Sigma) to detect tumor areas with high nuclei density by fluorescence. Sections were mounted on glass slides cover slipped using Fluoromount^™^ Aqueous Mounting Medium (Sigma). Images were obtained using a Nikon Ni-E. CD31^+^ vessels were quantified based on the fluorescence signal using ImageJ as previously described [26]. To ensure comprehensive analysis, multiple pictures of the tumor core were captured for each mouse. The quantification of hKi67, Iba1, and mCD31 was performed using several technical and biological replicates, with each dot on the boxplots representing an individual image obtained from each tumor. Number of images per mouse depended on the tumor size. Mouse sample size for hKi67 quantification was n = 2 for P3 CTR, n = 2 for P3 resected; n = 1 for T16 CTR and n = 3 for T16 resected. Mouse sample size for Iba1 and mCD31 was n = 4 for controls and n = 6 for resected.

## Results

### High subset of intracranial tumors derived as glioma PDOX models can be reliably detected and quantified by MRI, allowing for MRI-guided surgical resection

**Fig. 1:**
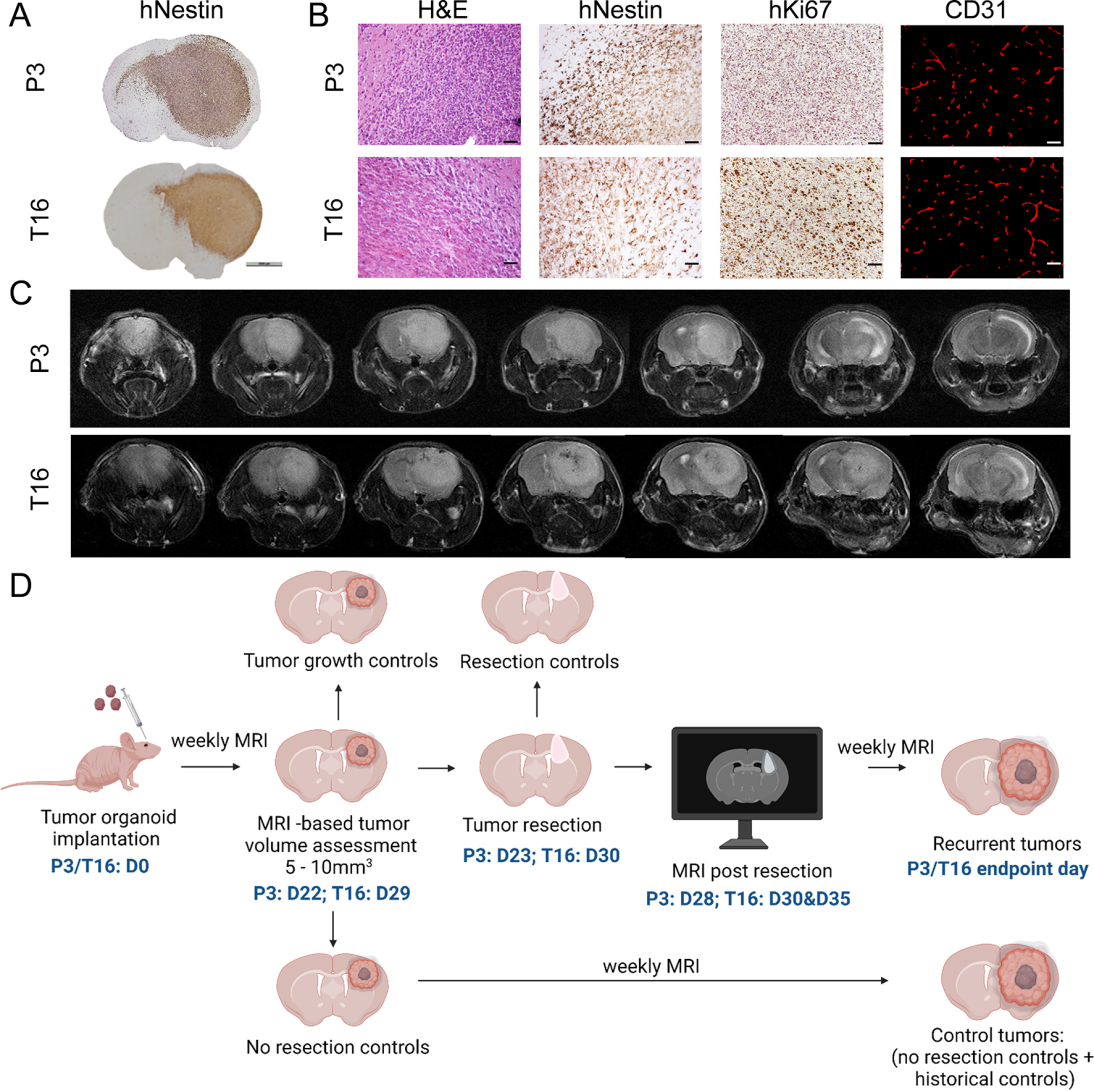
PDOX characteristics. (**A**) Histopathological features of intracranial tumors in PDOX models. Representative images are shown for the selected models at the endpoint of tumor growth. Human-specific Nestin staining highlights the overall tumor structure and invasion of tumor cells in the brain. Scale bar: 2 mm. (**B**) Hematoxylin and eosin (H&E) and human-specific Nestin staining highlight tumor cell density and invasion, while Ki67 marks actively proliferating cells. Mouse-specific CD31 staining shows aberrant blood vessels in the tumor core. Scale bars: 50 μm. (**C**) Tumor visualization by MRI. T2-weighted MRI images at the end point in the mouse head are shown for selected models, and representative slices are shown from anterior to posterior with tumors visible as a relatively brighter area in the brain. (**D**) Experimental setup of the resection protocol. Day 0 (D0) represents GBM organoid implantation. Tumor growth can be followed by MRI T2 from week 1 after implantation. The experimental window of tumor resection is highlighted together with the exact time points selected in this study for PDOX P3 and PDOX T16. Tumor regrowth was followed by MRI, and the mice were euthanized when the symptoms reached the endpoint. Additional controls applied in the study are presented. Created with BioRender.com

To investigate the possibility of clinically relevant resection of primary diffuse glioma tumors developed in mice, we took into account several parameters, including diffused tumor growth and invasiveness of the tumor cells in the mouse brain, tumor take, tumor development time and endpoints. PDOX models in our cohort represent high-grade diffuse gliomas, including IDH wild-type GBMs and IDH mutated astrocytomas (Table S1). In general, upon good organoid quality, PDOXs have tumor take of 100% and predictable tumor growth beyond generation 2–3 [23]. By implanting 6 organoids per mouse brain, the survival of mice can span between 36 and 394 days depending on the model. Out of 40 models examined by MRI, intracranial tumors of 36 models (90%) were detected by MRI, of which the detection level in 22 models (55%) allowed for reliable quantification of the 3D tumor volume (Table S1). Tumors can be detected starting from approximately 2 mm^3^ in size in the brain. Fast-growing tumors are detectable by T2-weighted MRI volumetric brain scans from as fast as two weeks after organoid implantation. For the majority of PDOXs, 1–2 months are needed to detect small tumors by MRI. Although histologically developed tumors show varying tumor volume, cell density and angiogenesis, diffuse growth and invasion to the cortex and via the corpus callosum is observed in all PDOXs [23, 26] (Fig. 1A, B).

To assess the surgical resection protocol, we selected two models representing IDH wild-type GBMs with varying survival times that can be reliably detected and quantified by MRI: PDOX P3 (survival: 42.5 ± 5.2 days) and PDOX T16 (survival:58.5 ± 5 days). Both models displayed histopathological GBM characteristics, such as marked invasion to the cortex and contralateral hemisphere as well as pronounced blood vessel disruption in the tumor core (Fig. 1A, B). Differences in survival arise from different proliferation indexes: PDOX P3 shows 54.7 ± 3.7% of Ki67^+^ cells, while PDOX T16 regularly shows 18.9 ± 5.8% of Ki67^+^ cells. The differences in tumor cell proliferation and survival times allowed us to verify the protocol timeframe and impact of the surgery in tumors with variable growth dynamics. PDOX P3 can be detected by T2-weighted MRI starting from weeks 2–3 after implantation and can reach > 100 mm^3^ at the endpoint (Fig. 1C). PDOX T16 is visible by T2-weighted MRI from weeks 3–4 after implantation, reaching > 100 mm^3^ at the endpoint (Fig. 1C). Based on the tumor growth characteristics, we estimated a timeframe of surgical resection between weeks 2 and 3 for PDOX P3 and between weeks 3 and 4 for PDOX T16, corresponding to tumors ranging between 2 and 10 mm^3^ (Fig. 1D). In summary, glioma PDOXs show key characteristics required to model surgical resection.

### Intracranial tumor mapping by MRI allows for preoperative neuronavigation planning of surgical resection

**Fig. 2:**
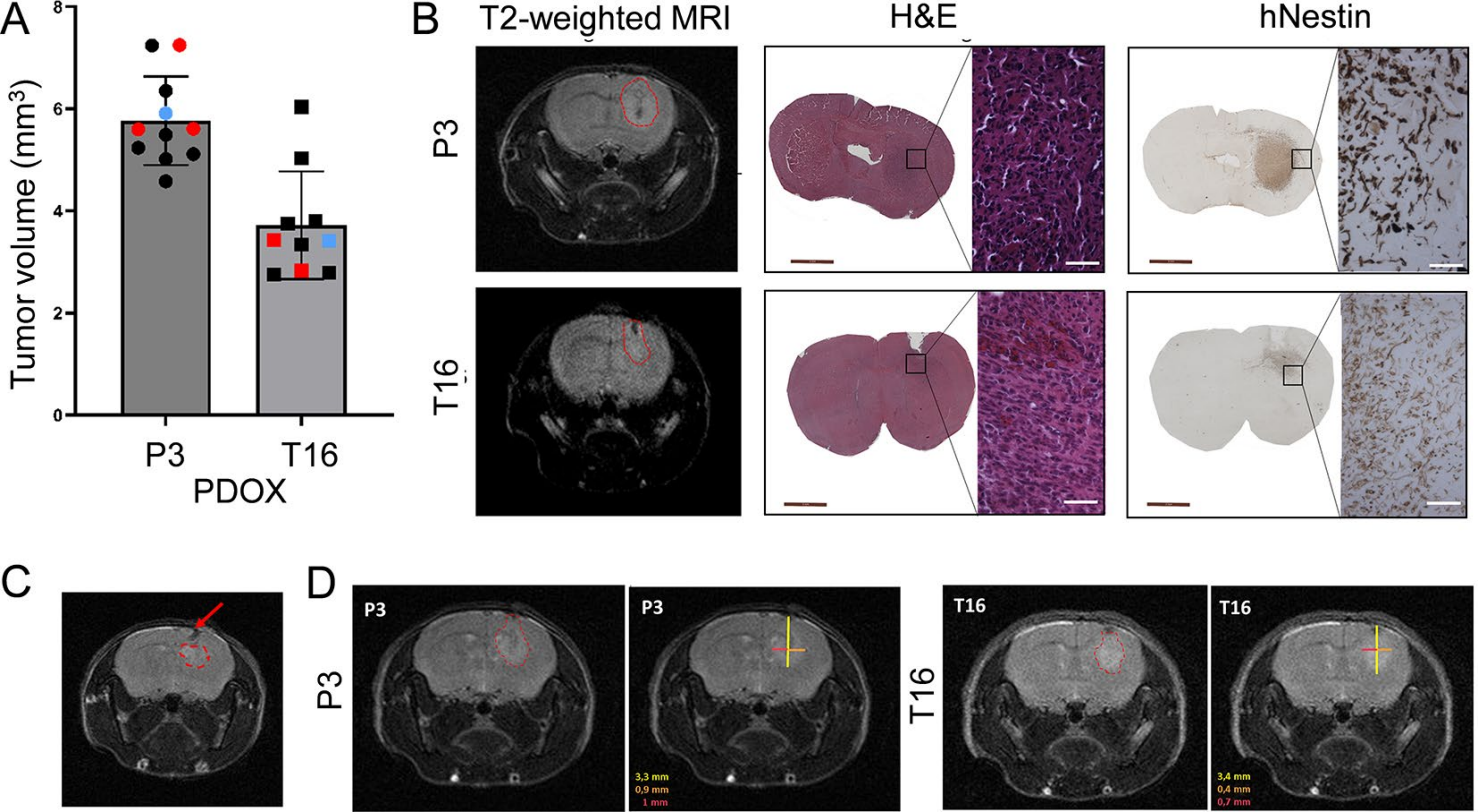
Preoperative MRI imaging for neuronavigation. (**A**) T2-weighted MRI-based quantification of tumor volumes one day prior to surgical intervention (mean ± SD). Control mice are highlighted: red - control mice without resection (i.e., tumor growth controls and no resection controls), blue – mice euthanized shortly after resection (i.e., resection controls). (**B**) T2-weighted MRI of PDOX P3 and PDOX T16 mice euthanized at the resection time without resection (i.e., tumor growth controls). Associated H&E and human-specific Nestin staining show the presence of tumor cells. Inserts highlight the presence of invasive tumor cells at the tumor borders. Scale bars: 2 mm (brown) and 50 μm (white). (**C**) A representative T2-weighted MRI showing the injection site of organoid implantation (red arrow) and the delineated tumor developed in the mouse brain (red dashed line). (**D**) Representative T2-weighted MRI of intracranial tumors in PDOX P3 and T16, one day prior to resection (left panels) and the associated measures to define the resection area (right panels). The resection was defined from the injection point with 3.3 mm of depth, 0.9 mm on the right side and 1 mm on the left side for the PDOX P3 tumor, and with 3.4 mm of depth, 0.4 mm to right side and 0.7 mm on the left side for the PDOX T16 tumor

We planned the resection protocol according to the characteristics of each PDOX model (Fig. 1D). Nude mice per model were implanted with GBM organoids (n = 11 for PDOX P3, n = 10 for PDOX T16) The implantation was performed following our standard procedure in the stereotactic frame [22]: (i) the scull bone was removed at the coordinates 2 mm right and 1 mm front from bregma, and (ii) 6 organoids were gently implanted in the right hemisphere 2 mm deep from the brain surface, aiming at the cortex. We further monitored tumor growth by T2-weighted MRI.Tumors were detectable in all animals starting from week 3 for PDOX P3 and week 4 for PDOX T16. Tumors were quantified one day before planned surgery based on T2-weighted MRI imaging, confirming an average tumor volume of 5,76 mm^3^ in PDOX P3 and 3,72 mm^3^ in PDOX T16 (Fig. 2A). To confirm the tumor size determined by MRI prior to resection, we euthanized one mouse per PDOX model without performing the resection protocol on the same day (i.e., Tumor growth controls, Fig. 1D). Histological analysis confirmed the presence of tumors in both PDOX models, corresponding to the MRI images (Fig. 2B). Human-specific Nestin staining revealed the presence of cells invading the corpus callosum and cortex at this stage of the tumor.

Next, we examined T2-weighted MRI sequences to define the tumor resection area following pre-operative neuronavigaiton principles. The injection site was identified based on the dark area representing the path of the needle during organoid implantation (Fig. 2C). The tumor area was delineated from the injection site, and tumor borders were identified manually (Fig. 2D). The thickness of the tumor was defined by the number of MRI slides with detectable tumor. In most cases, the tumor was visible on one slide of 1 mm thickness, 0.5 mm in the frontal part and 0.5 mm in the posterior part, which was applied to mark the extent of the resection. In summary, we show that T2-weighted imaging enables quantification of the intracranial growth of tumors in mice and detailed planning of the surgical resection coordinates.

### Successful intracranial Tumor resection in PDOXs following MRI coordinates

**Fig. 3:**
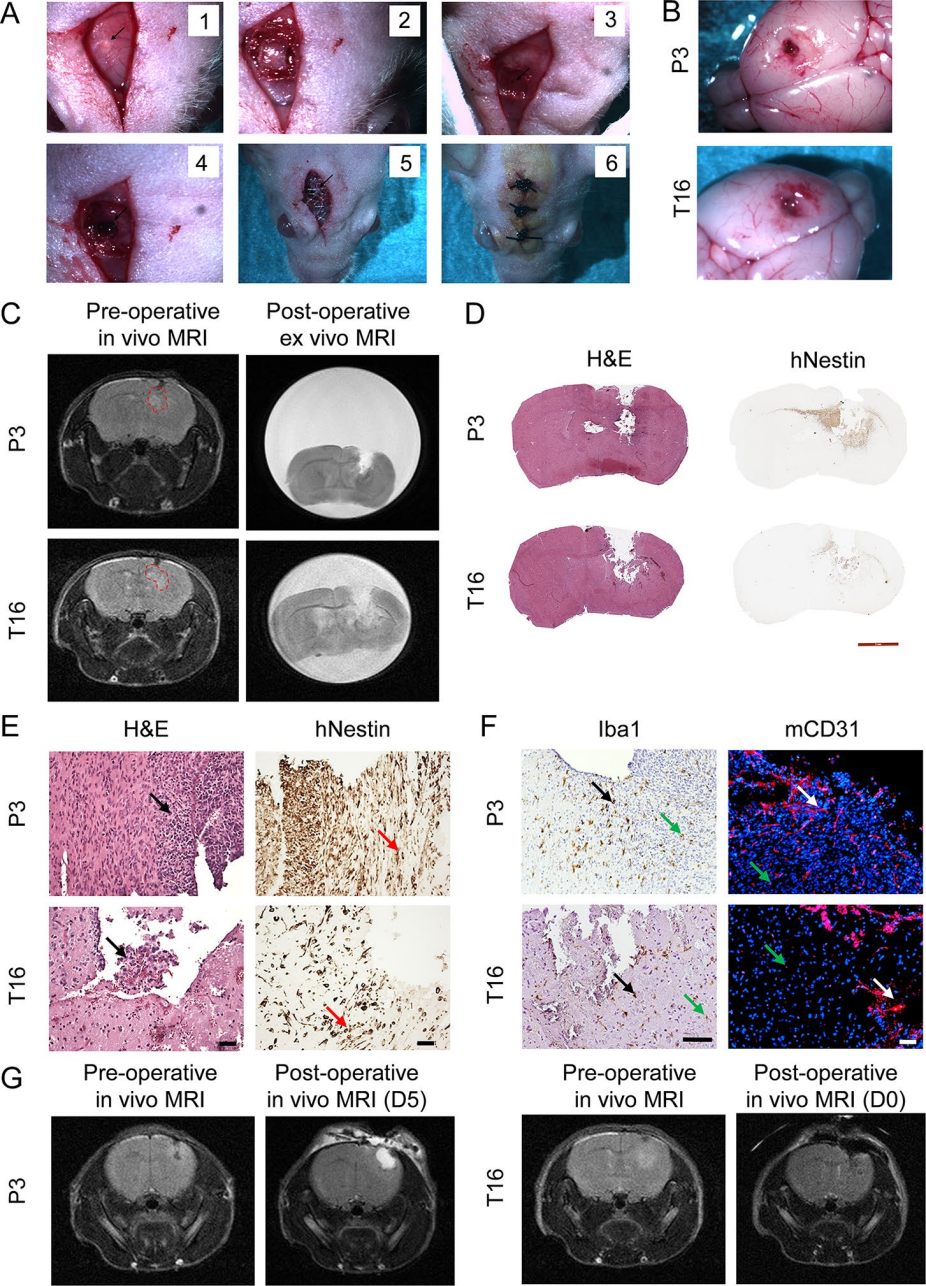
Intracranial tumor resection. (**A**) Resection protocol steps. Representative intraoperative images are shown for one nude mouse. (1) The skin incision and the hole covered by bone wax (from tumor implantation) were exposed (black arrow). (2) A square with approximately 3 mm sides was drilled. (3) The square of the skull was removed, and the hole of the GBM organoid implantation was identified (black arrow). (4) Resection area obtained after dura mater peeling and aspiration of tumor-containing tissue. (5) Polypropylene meshes (black arrow) were fixed with hystoacryl to replace the missing skull bone. (**B**) Ex vivo mouse brains extracted shortly after resection (i.e., resection controls) showing the resection area for PDOX P3 and PDOX T16. (**C**) Ex vivo T2-weighted MRI of PFA-fixed brains extracted shortly after tumor resection (right). No remaining tumor was detected by T2-weighted MRI. The corresponding preoperative in vivo MRI images are shown for comparison for each mouse (left). (**D**) H&E staining and human-specific Nestin staining in the brains extracted shortly after tumor removal confirm a significant resection area in the brain. Remaining tumor cells are present around the resection site and in the corpus callosum. Scale bar: 50 μm. (**E)** Magnified representative images of the resection area highlighting the presence of tumor cells directly around the resection area (black arrows) and tumor cells invading the corpus callosum (red arrows). Scale bar: 50 μm (**F**) Representative images showing mouse Iba^+^ microglia/macrophages with ameboid morphology (black arrows) and enlarged CD31^+^ blood vessels in the remaining tumor areas (white arrows). Iba1 + ramified microglia and small CD31^+^ blood vessels are visible in the areas of normal brain devoid of tumor cells (green arrows). Scale bar: 50 μm. (**G**) Representative T2-weighted MRI performed before (left) and after tumor resection (right): 5 days after resection for PDOX P3, directly after the surgery for PDOX T16. For PDOX P3, the image shows a bright area most likely representing a resection cavity filled with a liquid

We performed surgical resection on eight mice per model one day after the MRI scans. Following the principles of the neuronavigation, the resection area was delimited per mouse based on coordinates established from MRI imaging, and each surgery was performed under the stereotactic frame. To ensure sufficient analgesia, we applied subcutaneous injections of buprenorphine. Addition of adrenaline to the local anesthesia helped to reach vessels vasoconstriction and reduce bleeding during surgery. We applied the coordinates previously used as the injection site of the GBM organoids (Fig. 3A), identified as the burr hole in the scull previously filled with bone wax. After skin incision, we drilled a 3 mm side square and removed the skull to prepare the resection area and improve the visibility of the injection site (Fig. 3A). After opening the dura mater, we applied the resection coordinates and cut the tissue by slowly moving the Bonn Micro probes. In contrast to the Hamilton syringe used for implantation of GBM organoids, the Bonn probe is thin and sharp and allows precise cutting of the brain tissue. Destroyed tissue was further aspirated with a Pasteur pipette and the manual aspiration system. The mean time of the surgical procedure was 20 min per mouse. Of note, since the GBM tumor borders in PDOXs cannot be identified by eye, the MRI-based coordinates were the only measure allowing for a controlled resection. Removal of the tumor tissue according to the MRI coordinates was successful in all mice, with a well-visible resection cavity. To replace the missing skull bone, we placed polypropylene meshes and fixed them with hystoacryl. This ensured clinically relevant regrowth of the tumor with adequate intracranial pressure in the brain.

Since one mouse per model suffered from respiratory depression due to anesthesia shortly after the resection, we sacrificed these mice (i.e., Resection controls, Fig. 1D) to verify the extent of the resection by MRI and immunohistochemistry in extracted brains (Fig. 3B). The high-resolution T2-weighted MRI performed on the PFA-fixed brain ex vivo revealed tumor resection according to coordinates defined prior the surgery (Fig. 3C). The histological analysis further validated the presence of remaining tumor cells around the resection cavity and at the invasive front (red arrow) in the cortex and in the corpus callosum (Fig. 3D, E). These were accompanied by amoeboid Iba1^+^ microglia/macrophages and aberrant CD31^+^ blood vessels, reminiscent of the GBM-educated tumor microenvironment (Fig. 3F).

We further aimed to confirm the extent of tumor resection in remaining PDOXs. Since the first experiment was performed on PDOX P3, to avoid mouse suffering/loss, T2-weighted MRI scans were performed 5 days after resection. Three mice showed no residual tumor in the brain, and four mice showed growing tumors between 5 mm^3^ and 12 mm^3^ (Fig. 3G). Since MRI was not performed immediately after the surgery, we cannot determine whether this difference arose from a lesser extent of the surgery or faster tumor growth. Since mice supported well T2-weighted MRI shortly after the surgery, PDOX T16 mice were scanned directly after the surgery before the awakening of the mice and 5 days after the surgery (Fig. 3G). No remaining tumor was detected on MRI in the seven mice examined directly after the surgery, and only one mouse showed a small detectable tumor 5 days after the surgery (Table S2). In summary, we show an efficient protocol for the resection of diffuse tumors developed in mouse brains.

### Resection is ineffective to cure GBM in PDOXs as recurrent tumors regrow within the resection cavity

To assess mouse recovery and survival after surgical resection, we followed daily PDOX mice with resected tumors (seven per model) and control PDOX mice, which did not undergo surgical resection (i.e., No resection controls, Fig. 1D). All mice recovered after surgery. During the first 48 h of recovery, mouse body weight decreased on average between 5% and 10%. Animals presented few neurological symptoms, such as disorientation and circling. All symptoms disappeared 48 h after the surgery. Mice recovered their presurgery body weight and displayed normal behavior.

**Fig. 4:**
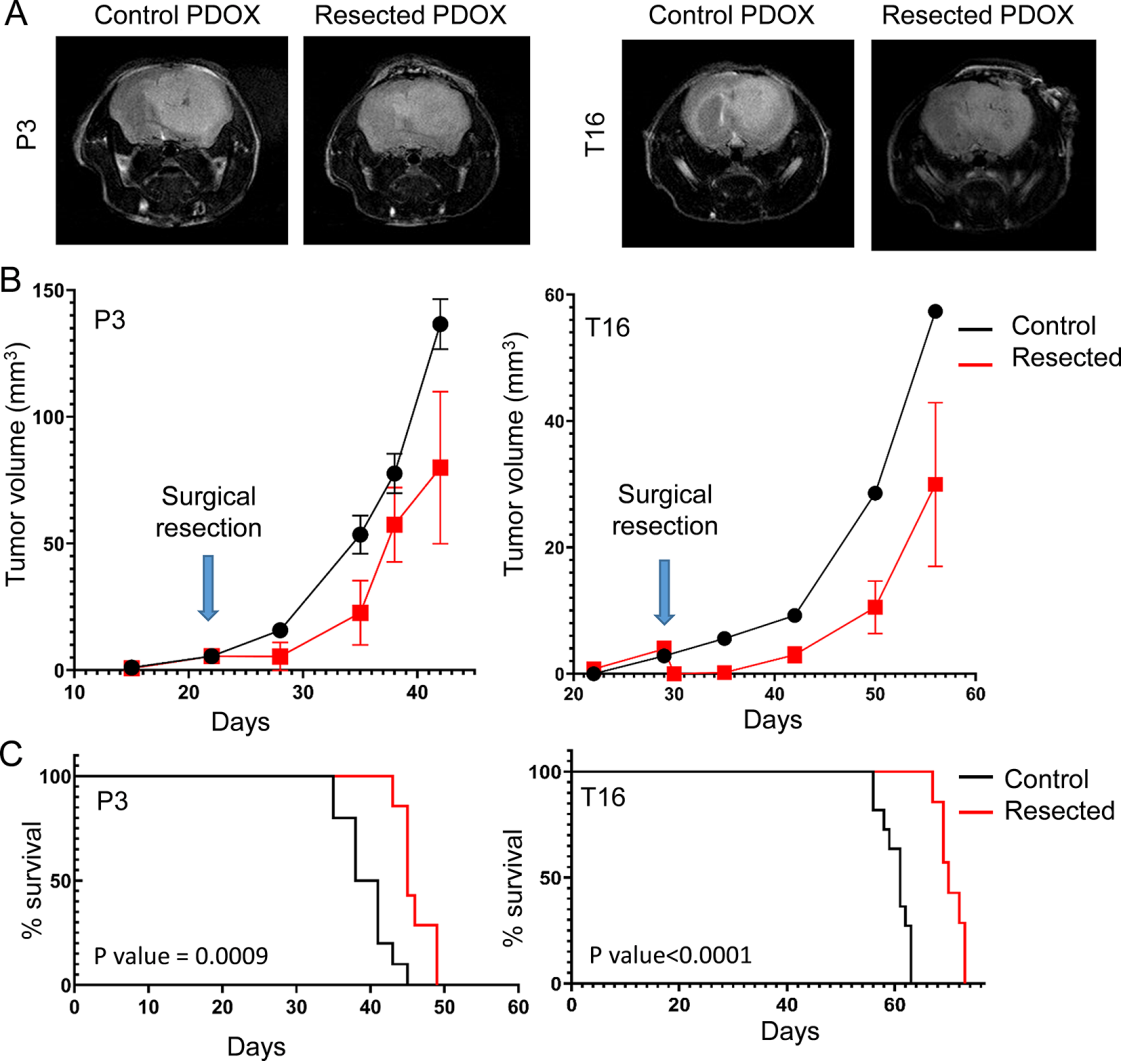
Tumor recurrence after surgical resection. (**A**) Representative T2-weighted MRI of a control PDOX brain and a resected PDOX brain showing similar tumors at the endpoint. MRI was performed before euthanasia. (**B**) MRI-based quantification of tumor growth over time (mean ± SD, resected group: n = 7 for both models, control group: n = 2 for P3 and n = 1 for T16. (**C**) Kaplan‒Meier curves of control PDOXs (P3: n = 8 historical controls and 2 control mice from the study, T16: n = 10 historical controls and 1 control mouse from the study) and resected PDOXs (n = 7) shown for PDOX P3 and PDOX T16. A median survival increase of 5.5 days was observed for PDOX P3 (p value < 0.0042) and 9 days for PDOX T16 (p value < 0.0001, long-rank test)

T2-weighted MRI scans revealed regrowth of the tumor in all PDOXs P3 and T16 that underwent tumor resection. All tumors recurred in in the resection cavity (Fig. 4A). Weekly MRI showed a decrease in tumor growth in both PDOX models after surgical resection (Fig. 4B). To further assess the impact of resection on mouse survival, we compared PDOXs that underwent resection to the historical control groups (n = 10 for P3, n = 11 for T16 per model including study controls and historical groups) (Fig. 4B). Survival of mice that underwent resection was significantly prolonged. For PDOX P3, the mean survival increased from 39,5 days for the control group to 45 days for resected mice (16%, p value = 0.0009). PDOX T16 showed an increase in the mean survival from 61 days for control mice to 70 days for the resected group (14,8%, p value <0.0001).

**Fig. 5:**
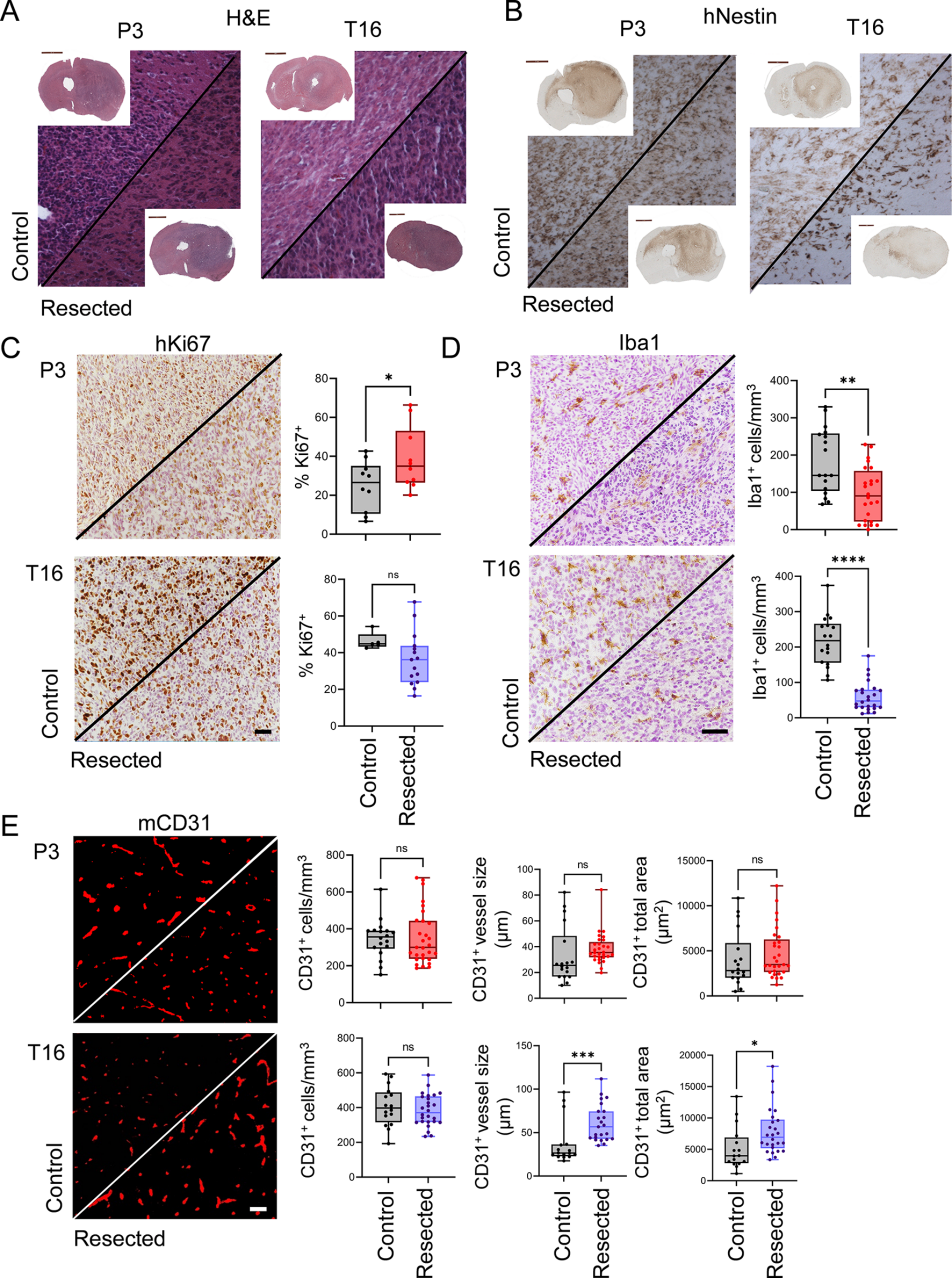
Histopathological assessment of resected tumors. (**A, B**) Representative H&E and human-specific Nestin stainings are shown for tumors at the endpoint in control and resected PDOXs. No histopathological differences were observed in recurrent tumors after resection. Scale bar: 50 μm. (**C**) Representative human-specific Ki67 staining highlights proliferative cells. Scale bar: 50 μm. (**D**) Representative Iba1 staining shows a decreased density of mouse-derived microglia/macrophages in resected PDOX tumors at the endpoint compared to controls. Scale bar: 50 μm. (**E**) Representative mouse-specific CD31 staining depicts a similar density of mouse-derived blood vessels with increased size in resected PDOX T16 compared to its control counterparts at the endpoint. Scale bar: 50 μm. For the entire figure: *p < 0.05, **p < 0.01, ***p < 0.001, two-tailed Student’s t test. Individual data points correspond to images obtained in different regions of tumors in several mice per each experimental group. In box plots, the box limits indicate the 25th and 75th percentiles, and centerlines show the medians; whiskers represent the minimal and maximal observed values

We next assessed the histopathological features of the tumors that recurred after resection. We confirmed the presence of the tumors in the initial location and did not reveal any obvious structural differences between control and resected tumors (Fig. 5A, B**)**. Recurrent tumors showed high tumor cell density in the right hemisphere (implantation site) as well as invasion to the cortex and corpus callosum. While Ki67 staining revealed an increase in the proliferation index in recurrent tumors in PDOX P3 compared to historical controls, no difference was observed in PDOX T16 (Fig. 5C**)**. Interestingly, while ameboid-shaped Iba1^+^ tumor-associated microglia/macrophages were detected in the tumor core of both control and recurrent tumors, both models showed decreased density in resected tumors (Fig. 5D**)**. CD31 staining displayed a similar density of blood vessels between the control and resected tumors, although the average vessel size was significantly higher in the resected PDOX T16 tumors than in the historical controls (Fig. 5E). In summary, we show that surgical resection of PDOXs developed in nude mice leads to prolonged survival and tumor recurrence.

## Discussion

In vivo modeling of gliomas in animals has long been challenging. Syngeneic and genetically engineered mouse models show limited resemblance to human gliomas, whereas subcutaneous and orthotopic xenografts based on human glioma cell lines do not faithfully recapitulate all characteristics of patient tumors [5]. In particular, recapitulation of diffuse cell growth in the normal brain has been limited in the majority of in vivo glioma models, restricting their use for clinically relevant surgical interventions [27]. We have previously shown that intracranial implantation of organoids derived from human gliomas, in particular GBMs, allows for efficient development of PDOX models in immunodeficient mice [22, 23, 26]. Developed human tumors are well integrated in the mouse brain structure and recapitulate various histopathological characteristics of human tumors, including diffuse growth in the brain (i.e., invasion), necrosis, pseudopalisading hypoxia and microvascular proliferation. PDOXs faithfully preserve the genetic, epigenetic, transcriptomic, metabolomic and stem cell-like profiles of human gliomas [23, 28–31]. Human tumor cells further reciprocally interact with the mouse brain to create a clinically relevant tumor microenvironment [32]. While longitudinal PDOXs derived from the same patient at different time points of the clinical treatment protocol allow for monitoring long-term tumor evolution, preclinical testing requires direct treatment in mice. We have shown that PDOX models are amenable for various therapeutic intervention studies due to efficient tumor take, stable tumor growth and endpoints, and detectability by MRI [23, 33, 34]. Here, we further aimed to test whether surgical resection of GBM PDOX tumors can be performed in a repeatable manner in mouse brains with clinically relevant outcomes.

Tumor resection is a key step in the clinical practice of aggressive brain tumors; hence, it is rarely recapitulated in preclinical models. To date, the majority of treatments are being tested on small tumors developing in animals at early stages. Surgery in small rodents is more challenging due to the small size of the organs, especially in mice. To date, tumor resection protocols in mouse brains are rare and do not yet involve regular application of PDOX models based on unmodified patient-derived tumor cells. Here, we implemented a new MRI-guided procedure for brain tumor resection in immunodeficient mice. We show that mice can support two consecutive surgeries, the first surgery for GBM organoid implantation, followed by the second intervention for tumor resection a few weeks later. T2-weighted MRI imaging allows for detailed monitoring of tumor growth and detailed planning of the tumor resection coordinates. We show that tumor resection leads to clinically relevant outcomes, including recurrence of tumors from the remaining invasive tumor cells and prolonged survival.

MRI imaging is a fundamental step in the preoperative diagnosis and mapping of brain tumor patients and planning of surgery. In the clinical setting, T2-weighted imaging allows detecting the tumor core and is supported by T1-weighted imaging with contrast for visualization of tumor areas with the leaky blood‒brain barrier. We have previously shown that T2-weighted imaging allows for the detection and quantification of intracranial GBM growth in PDOXs in mice, while detection by T1-weighted MRI with contrast is limited, most likely due to small tumor size and restricted size of hypoxic and necrotic areas with limited blood‒brain barrier leakage [22, 23, 26]. This is improved in nude rats, where tumors reach higher volumes at endpoints [23]. In our current cohort, the majority of models are detectable by MRI, and only very invasive PDOXs with low tumor cell density are invisible. Similar to the patient tumors, invasive tumor areas in each model are not detectable, allowing for a clinically relevant setup of surgical resection based on MRI-guided coordinates. Having demonstrated the consistent quantification of tumor volumes in 55% of the models within our current cohort throughout the growth process, our procedure can be significantly scaled up to other PDOXs. The surgical approach can be thus envisioned for application in combination with personalized drug efficacy studies in the selected tumor types.

Importantly, our approach is applicable to patient-derived models without the need for additional genetic modification of tumor cells. This is an improvement in comparison to previous reports presenting protocols following ocular or fluorescence-guided resection on orthotopic brain tumors developed in mice based on cell lines (U87, GL261) previously modified to express fluorescent and/or bioluminescent proteins [35–37]. Moreover, while the majority of cell line-derived orthotopic tumors create visually distinguishable tumor ‘’lumps’’, tumors developed from GBM organoids are well integrated in the mouse brain, mostly undistinguishable by eyes or under binocular. MRI also has an advantage over 18 F-FET PET/CT-based follow-up of resected tumors, which is limited only to tumor models showing high uptake of the tracer in vivo [19]. As intraoperative MRI is not practical for mice, MRI-guided coordinates established prior to surgery are needed for precise, clinically relevant resection.

Although not curative, the extent of surgical resection in GBM patients has been linked to prolonged survival [11, 38]. Here, we show that resection of GBM tumors in mice also leads to prolonged survival with inevitable recurrence. Following our animal experimentation protocol, to avoid unnecessary use of animals, we compared the survival of resected mice to that of our historical controls. This was possible due to the high reproducibility of tumor growth in the selected PDOX models. As we did not perform additional treatments, mock surgeries were not necessary. These factors should be considered if the systemic response to injury may influence the experimental outcome [16]. The surgical procedure did not reveal major technical issues, and the majority of mice survived after two consecutive brain surgeries. In this study, only two mice (one per model) suffered from respiratory depression, most likely due to anesthesia during surgery. Another cause may be linked to the use of ketamine and xylazine, which could have induced hypotonia and consecutive hypoxemia, hypercapnia and acidosis.

Noticeably, the resection protocol was not curative, and residual tumor cells that remained in the mouse brain led to tumor regrowth in the initial position along the resection cavity. This is in line with the clinical situation, as the majority of GBM tumors typically recur locally, with recurrent tumors extending from the resection cavity of the initial surgery [39]. The resection time point and consecutive time of recurrence were linked to the PDOX model used, and more proliferative PDOX P3 recurred faster than less proliferative PDOX T16. Surgical resection in the PDOX models led to a median survival benefit of 6 days for PDOX P3 and 9.5 days for PDOX T16. This is comparable to the survival benefits reported for cell line-based xenografts in mice (GL261 4–5 days [16, 35, 36], U87 8–10 days [37, 40]), while a > 20 day survival benefit was reported in GBM PDOX P3 developed in nude rats [21]. The difference likely arises from a different time point possible for the surgery (week 4 after implantation) and larger tumor volumes that can be achieved in rats. Similar to P3 tumors in nude rats, we observed an increased proliferation index in resected tumors; however, this difference was not confirmed in the second PDOX model (T16).

We did not observe major changes in the structure of the recurrent tumors in resected mice. Our data are in line with observations in recurrent GBM patient tumors that show similar histopathological features and overall structure [41, 42]. Recurrent tumors showed similar invasion to the cortex and corpus callosum as well as the mouse-derived components of the tumor microenvironment. While the density of tumor-associated microglia/macrophages was observed in the two models, only a minor increase in the size of blood vessels was observed in the recurrent tumor of PDOX T16. This is in contrast to the findings in nude rats, where significantly increased blood vessel area and increased density of macrophages were reported in tumors that regrew after resection [19, 21]. More data are needed to understand whether such differences are patient- and/or model dependent. Moreover, the composition of the tumor microenvironment may depend on the tissue collection time point after the surgery. Our protocol allowed for mouse follow-up until the endpoint, potentially allowing for a longer time for the tumors to recover from the surgical intervention.

Categorization of the extent of the surgery, regularly performed in patients, may be challenging in mice due to limited contrast enhancement. Based on the postoperative T2-weighted MRI imaging and histopathological analysis of resected tumors, we estimated that we reached “near total” (80–90% tumor core removed) to “complete/gross total” (approximately 100% tumor core removed) extent of surgery. This parameter can be adjusted according to the protocol needs by adapting MRI-based coordinates to achieve less invasive resection, i.e., the “subtotal” level (< 80%). In this context, further optimizations of tumor detection by fluorescence may include ALA-5, a fluorescent agent that is regularly used to visualize brain tumor cells during surgeries in glioma patients. Potentially it could allow reaching the ‘’suboptimal’’ resection (extended to/beyond tumor borders). However, it remains to be seen whether blood‒brain barrier leakage in GBM PDOX models is sufficient to reach optimal staining for tumor cells with ALA-5, as ALA-5 is impermeable though the intact blood‒brain barrier [20, 43].

In summary, we present an efficient and clinically relevant protocol for the resection of GBM tumors using PDOX models developed in mice. In the future, this model can be combined with standard-of-care treatment and/or for testing novel treatment strategies in a larger cohort of PDOX models with varying molecular backgrounds. While immunocompetent models are needed for testing modulations of the immune system linked to surgery [16], PDOX models may be beneficial for interventions targeting tumor cells. For example, resected GBM PDOXs may be applied as a valuable tool for the assessment of the molecular evolution of tumors and phenotypic plasticity directly upon treatment [44]. In particular, it may be relevant for testing novel modalities requiring local delivery to the resection cavity and/or drugs aimed at targeting invasive cells shortly after surgery. The model could also be applied to develop MRI- and/or PET-based protocols for the visualization of invasive cells present in the brain not available for surgical resection and image-guided surgery protocols.

## Conclusions

In conclusion, our work demonstrates the feasibility of replicating surgical interventions in preclinical brain cancer models. We have successfully performed intracranial surgeries in GBM PDOX models, which accurately mimic the diffuse tumor growth observed in mouse brains. Similar to patient’s clinical protocols, the utilization of MRI enables the evaluation of tumor growth before and after the surgery, as well as facilitates the planning of surgical coordinates.

### Electronic supplementary material

Below is the link to the electronic supplementary material.

**Supplementary Material 1**: Table S1. Characteristics of the glioma PDOX model cohort

**Supplementary Material 2**: Table S2. Quantification of the tumor volumes after surgical resection

## Data Availability

PDOX models and molecular data are available via Patient Derived Cancer Models Finder (https://www.cancermodels.org/) and are part of the EuroPDX consortium collection (www.europdx.eu).
