# Supplementary material for: Magnetic resonance imaging-guided intracranial resection of glioblastoma tumors in patient-derived orthotopic xenografts leads to clinically relevant tumor recurrence
Source: BMC Cancer. 2024 Jan 2;24:3. doi: 10.1186/s12885-023-11774-6 (PMC10763155; doi:10.1186/s12885-023-11774-6)
Supplement: Supplementary file 1 — Supplementary Material 1: Table S1. Characteristics of the glioma PDOX model cohort [file 12885_2023_11774_MOESM1_ESM.pdf]

Table S1. Characteristic of the glioma PDOX model cohort.
Patient age, sex and diagnosis are provided.
For PDOX models average survival was calculated based on different batches, generations (G>2) and mouse strains.

**Model**	**Matched Patient information**					**PDOX characteristics**		
PDOX model	Patient code	Age at collection	Sex	Histological diagnosis	IDH mutation	PDOX generation reached	PDOX survival time (days)	Detection by MRI
P3	BER0003	64	Male	GBM Grade IV		>G10	42 +/- 5.2	detectable, quantifiable
P8	BER0008	64	Female	GBM Grade IV		>G10	52.5 +/- 3	detectable, quantifiable
P13	BER0013	NA	Female	GBM Grade IV		>G10	35 +/- 2.5	detectable, non quantifiable
T16	LIH0016	52	Female	GBM Grade IV		>G10	58.5 +/- 5 (>G7)	detectable, quantifiable
T101	LIH0101	60	Male	GBM Grade IV		G7	106 +/- 5.5	detectable, non quantifiable
T158	LIH0158	70	Female	GBM Grade IV		G5	68 +/-1.6	detectable, quantifiable
T185	LIH0185	76	Female	GBM Grade IV		G6	89 +/- 10	detectable, non quantifiable
T186	LIH0186	34	Male	Anaplastic Oligodendroglioma Grade III	yes	G9	159 +/- 43	detectable, non quantifiable
T188	LIH0188	68	Male	GBM Grade IV		G6	63 +/- 3.9	detectable, quantifiable
T192	LIH0192	42	Female	GBM Grade IV		G6	71 +/- 17.8	detectable, non quantifiable
T226	LIH0226	61	Male	GBM Grade IV		G5	127 +/- 4.9	detectable, non quantifiable
T233	LIH0192	42	Female	GBM Grade IV		G5	148 +/- 7.8	non detectable
T238	LIH0238	42	Male	GBM Grade IV		G3	100 +/- 8.8	detectable, non quantifiable
T239	LIH0239	80	Male	GBM Grade IV		G3	97.5 +/- 2.3	NA
T251	LIH0192	43	Female	GBM Grade IV		G7	65.2 +/- 1.5	detectable, non quantifiable
T281	LIH0281	51	Female	GBM Grade IV		G4	126 +/- 6.1	detectable, quantifiable
T304	LIH0304	53	Male	GBM Grade IV		G7	89 +/- 4.5	detectable, quantifiable
T331	LIH0331	84	Male	GBM Grade IV		G4	116 +/- 8.1	detectable, quantifiable
T341	LIH0337	75	Female	GBM Grade IV		G4	47.2 +/- 0.4	NA
T347	LIH0347	41	Male	GBM Grade IV		G4	125.6 +/- 5.6	detectable, non quantifiable
T356	LIH0281	52	Female	GBM Grade IV		G5	203.6 +/- 19.7	detectable, quantifiable
T361	LIH0361	69	Female	GBM Grade IV		G5	79 +/- 0	detectable, quantifiable
T363	LIH0363	84	Female	GBM Grade IV		G3	140 +/- 0	non detectable
T367	LIH0367	69	Male	GBM Grade IV		G3	168 +/- 0	detectable, quantifiable
T384	LIH0384	50	Female	GBM Grade IV		G4	202 +/- 6.9	non detectable
T386	LIH0386	51	Male	GBM Grade IV		G4	104.1+/- 6.8	detectable, quantifiable
T394	LIH0394	45	Female	GBM Grade IV	yes	G5	68.56 +/- 0.9	detectable, quantifiable
T407	LIH0394	45	Female	GBM Grade IV	yes	G6	64 +/- 4.3	detectable, quantifiable
T434	LIH0304	54	Male	GBM Grade IV		G6	43.20 +/- 0	detectable, quantifiable
T470	LIH0347	42	Male	GBM Grade IV		G4	78.33 +/- 15.3	detectable, quantifiable
T476	LIH0476	75	Male	GBM Grade IV		G4	66 +/- 0	detectable, non quantifiable
T515	LIH0515	46	Male	Anaplastic Oligodendroglioma Grade III	yes	G5	393.8 +/-5 5.6	detectable, quantifiable
T591	LIH0384	52	Female	GBM Grade IV		G3	110 +/- 16	detectable, quantifiable
T744	LIH0744	69	Male	GBM Grade IV		G3	100 +/- 0	detectable, quantifiable
T756	LIH0556	46	Male	GBM Grade IV	yes	G5	77.9 +/- 1.4	detectable, quantifiable
T772	LIH0615	54	Female	GBM Grade IV		G4	107 +/- 0	detectable, non quantifiable
T784	LIH0784	56	Male	GBM Grade IV		G4	138 +/- 2	detectable, non quantifiable
T797	LIH0797	55	Female	GBM Grade IV		G3	119 +/- 34.5	detectable, quantifiable
T831	LIH0831	51	Female	GBM Grade IV		G3	70 +/- 0	NA
T832	LIH0831	51	Female	GBM Grade IV		G3	138.5 +/- 14.5	non detectable
T841	LIH0841	38	Male	GBM Grade IV		G3	49.2 +/- 1.1	NA
T861	LIH0841	38	Male	GBM Grade IV		G2	157.2 +/- 3.9	detectable, non quantifiable
T899	LIH0609	75	Female	GBM Grade IV		G2	70.7 + /- 2.6	NA
T905	LIH0609	76	Female	GBM Grade IV		G3	91 +/- 1.7	detectable, non quantifiable
T1053	LIH1053	62	Male	GBM Grade IV		G2	98.7 +/- 0.5	NA
T1070	LIH0784	60	Male	GBM Grade IV		G1	254.2 +/- 36.2 (G1)	detectable, quantifiable
