# Supplementary material for: Magnetic resonance imaging-guided intracranial resection of glioblastoma tumors in patient-derived orthotopic xenografts leads to clinically relevant tumor recurrence
Source: BMC Cancer. 2024 Jan 2;24:3. doi: 10.1186/s12885-023-11774-6 (PMC10763155; doi:10.1186/s12885-023-11774-6)
Supplement: Supplementary file 2 — Supplementary Material 2: Table S2. Quantification of the tumor volumes after surgical resection [file 12885_2023_11774_MOESM2_ESM.pdf]

Table S2. Quantification of the tumor volumes after surgical resection

	P3		T16		
Tumor volume (mm3)	1 day before resection	5 days after resection	1 day before resection	Directly after resection	5 days after resection
mouse 1	5,235	11,482	2,785	0	0
mouse 2	5,016	8,087	6,037	0	1,392
mouse 3	6,352	12,884	3,338	0	0
mouse 4	5,493	0	5,035	0	0
mouse 5	5,112	0	3,756	0	0
mouse 6	7,239	5,617	3,796	0	0
mouse 7	4,578	0	2,756	0	0
